# Standardization of the functional syndesmosis widening by dynamic U.S examination

**DOI:** 10.1186/2052-1847-5-9

**Published:** 2013-05-02

**Authors:** Omer Mei-Dan, Mike Carmont, Lior Laver, Meir Nyska, Hagay Kammar, Gideon Mann, Barnaby Clarck, Eugene Kots

**Affiliations:** 1 Department of Orthopaedics, Division of Sports Medicine, University of Colorado School of Medicine, Aurora, Colorado; 2 Princess Royal Hospital, Telford, UK; 3 Department of Orthopaedic Surgery, Sports Medicine Unit, “Meir” University Hospital, Kfar-Saba, Israel; 4 Department of Orthopaedic Surgery, Division of Sports Medicine, Duke University, Durham, NC, USA; 5 Ribstein Center for Sport Medicine Sciences and Research, Wingate Institute, Netanya, Israel; 6 North Shore Hospital and Auckland Radiology Group, Auckland, New Zealand; 7 Department of Radiology, Musculoskeletal and Invasive Radiology Unit, “Meir” University Hospital, Kfar-Saba, Israel; 8 Department of General Surgery, “Meir” University Hospital, Kfar-Saba, Israel

**Keywords:** Syndesmosis, Ultrasound, Dynamic examination, External rotation

## Abstract

**Background:**

Dynamic US examination is a convenient, accurate, inexpensive and reproducible diagnostic tool for assessing the integrity of the distal tibiofibular syndesmosis in ankle injuries. However normal values for physiological functional widening of the anterior tibiofibular clear space in healthy subjects has yet to be determined. The purpose of this study was to determine normal values for the syndesmosis clear space on ultrasound examination.

**Methods:**

We evaluated 110 healthy subjects. A dynamic U.S examination was performed in neutral (N), forced internal rotation (IR) and external rotation (ER) of the ankle. In each position the anterior tibiofibular clear space was measured at the level of the anterior inferior tibio-fibular ligament (AITFL). Height and calf length were also recorded. Results were analyzed in relation to age, activity, dominant leg and gender.

**Results:**

Mean age was 32 years (range 16–60). There were 59 males and 51 females. 60% were professional athletes. Mean height was 173 cm (range 149–192). Functional Mean position measurements for clear space opening were: N=3.7mm, IR=3.6mm and ER=4.0mm. In younger men and women the clear space was significantly wider in neutral (Men: Y=3.8, O=3.4 \ Women: Y=3.8, O=3.4) and with rotational force application (Men ER: Y=4.1, O=3.6 \ Women ER: Y=4.1, O=3.8) compared to older subjects (p<0.05). There was no correlation with activity, height or the leg length.

Females had a higher syndesmosis widening ratio (ER/N) under stress than males (p<0.01) this tended to occur more commonly in active subjects.

**Conclusions:**

Normal values for the syndesmosis clear space on ultrasound examination were determined as 3.78mm in neutral, 3.64mm in internal rotation and 4.08mm in external rotation. The clear space was shown to decrease with age both as an absolute measure and when rotational stresses are applied. Females tend to have a larger clear space and a greater functional widening.

These findings provide a useful reference for radiologists and sports physicians when performing ultrasound assessment of ankle syndesmotic injuries and we encourage use of this modality.

## Background

Syndesmotic or “high ankle” sprains comprise 1% to 20% of all ankle sprains [1,2]. These injuries are difficult to diagnose when there is no frank diastasis or unstable fracture. Delayed diagnosis of a high ankle sprain results in a longer recovery time [3] and so may be termed latent. Failure to identify or stabilize a syndesmotic injury may predispose to joint instability and abnormal loading and lead to post-traumatic arthritis [4].

Frequently imaging is obtained only after an ankle sprain fails to settle with conservative therapy. Syndesmosis opening has been shown using plain radiographs and fluoroscopy with stress examination [5] however both require relatively cumbersome equipment, involve radiation exposure and are susceptible to variation in projection and accuracy. Magnetic resonance imaging may be considered to be the gold standard for detecting anterior inferior tibio-fibula ligament (AITFL) injury but is expensive, not readily available and only provides information during static positioning. Ultrasound examination has recently been shown to be highly sensitive and specific for AITFL injury in patients with normal radiographs when compared to MRI [6]. In addition the relative convenience, accessibility, safety and the low cost of ultrasound examination makes it a potentially popular imaging modality. Dynamic ultrasound may allow the functional stability of the ankle joint to be determined providing valuable information to guide treatment. The normal dimensions of the anterior clear space and how this varies with loading have yet to be determined on ultrasound examination. These values will aid in the diagnosis and may aid management decisions for high ankle sprain injury.

The aim of this study was to establish normal values for the width of the clear space opening in the neutral position and how this width changes with rotational loading in normal subjects on ultrasound examination. We also aimed to determine factors, which may predispose to anterior clear space opening and AITFL laxity in the normal population, how this width changes with rotational loading and to determine predisposing factors which lead to increased opening.

## Methods

We evaluated 110 healthy subjects all of whom gave informed consent for the study. : “Meir” University Hospital, Kfar-Saba, Israel ethics approval was obtained prior to recruitment. Ultrasound examination of both ankles was performed in all subjects by an experienced musculoskeletal radiologist (XX) and reviewed by a sports physician, using a 7.5-12 MHz linear transducer HDI 5000 SonoCT ATL (Philips, Andover, Massachusetts). Ultrasound images were obtained to visualize the syndesmosis landmarks, recorded and printed. The printed images were re-evaluated by the primary investigators. If a previous injury was reported or suspected due to a thickened, torn, scarred or deficient AITFL, the subject’s relevant ankle was excluded from the study.

**Figure 1:**
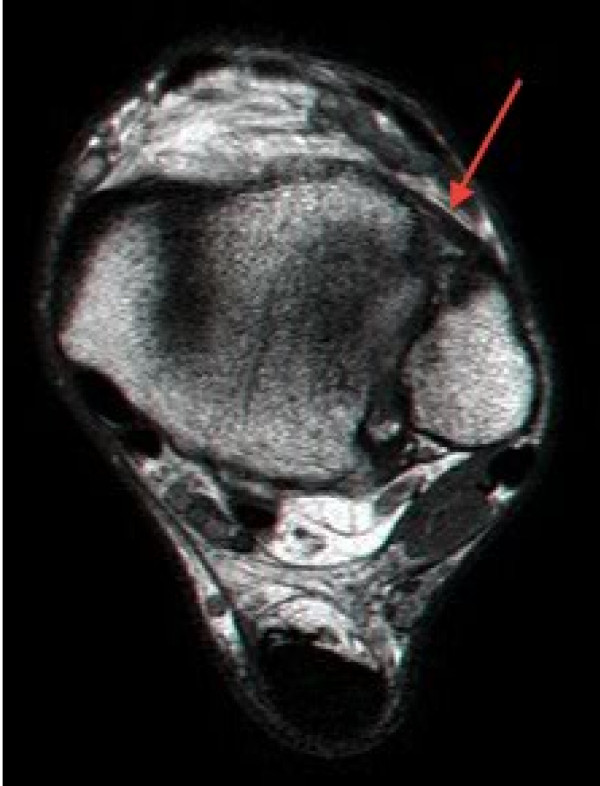
**An axial T1 Turbo Spin Echo magnetic resonance (MR) image, above joint level, where ultrasound measurements are taken.** Note the borders of the healthy Antero Inferior Tibio-Fibular Ligament (AITFL) marked with a red arrow.

**Figure 2:**
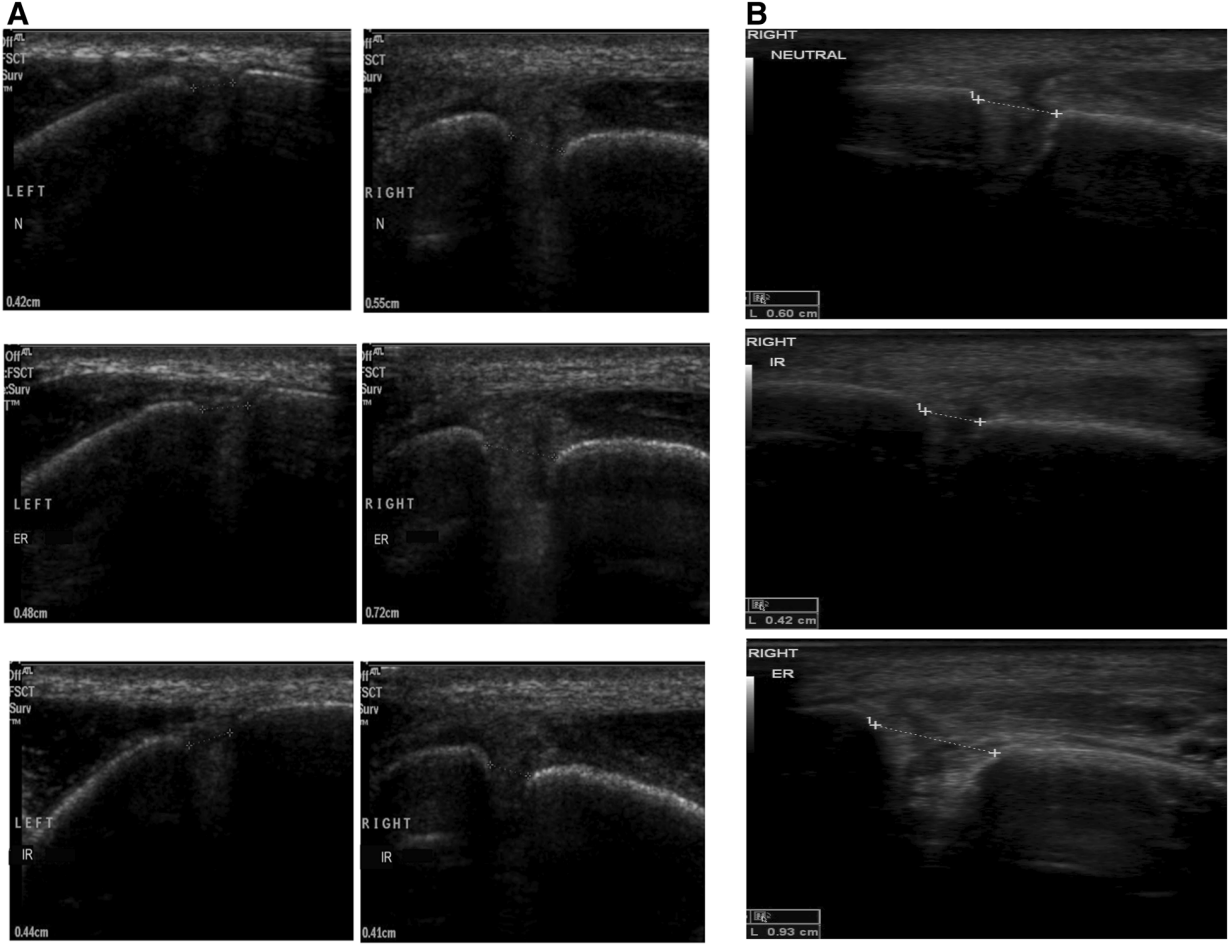
**Dynamic Ultrasound views of the Left clear space with healthy AITFL and the Right clear space with torn AITFL, of the same patient. A**: The healthy ligament is seen connecting the tibia to the fibula and with only a minimal measured clear space opening under stress (ER=External Rotation, IR=Internal rotation, N=Neutral). The right torn side shows a marked clear space opening under stress (ER) as there is no continuous ligament to stop separation of the tibia and fibula. Application of Internal Rotation stress tightens the joint, bringing the tibia and fibula closer to each other, in the absence of the AITFL. **B**: Dynamic Ultrasound views of a Torn AITFL which presents with widened clear space. (ER=External Rotation, IR=Internal rotation, N=Neutral). The injured syndesmosis shows a marked clear space opening under stress (ER) as there is no continuous ligament to stop separation of the tibia and fibula. Application of Internal Rotation stress tightens the joint, bringing the tibia and fibula closer to each other, in the absence of the AITFL.

**Figure 3:**
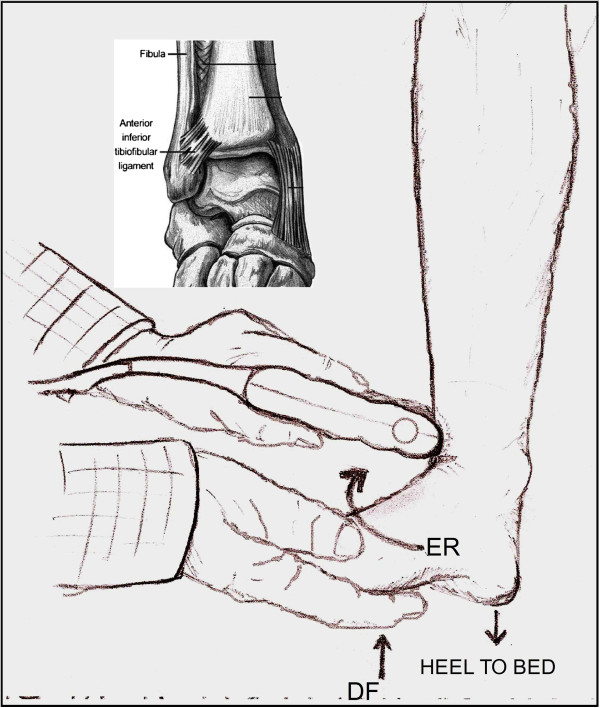
**Dynamic U.S examination of the AITFL.** In order to stress the lower syndesmotic joint into an External Rotation position the examiner is holding the foot and moving it towards external rotation and slight dorsiflexion of the ankle (see arrows). As in this diagram when the examination is performed by a single U.S operator the patient is asked to gently press his heel towards the bed to stabilize the ankle for stress maneuvers. The anatomy of the AITFL is also seen.

Examination technique was standardized as described by Mei-Dan et al. [4]. The patients were examined in the supine position (with the knee flexed to 90 degrees and the foot flush to the examination table). Anterior, lateral and medial compartments were evaluated to exclude previously injured joints. The syndesmosis was imaged in the transverse plane via the anterior approach. The AITFL was visualized for continuity and contour at 1cm proximal to the joint line just above the distal tibial anterior cortex where the ligament is thickest. (Figure 1) The distance between the tibia and fibula (clear space) was measured at the deep border of the ligament. Dynamic ultrasound examination was then performed using external and internal rotations of the foot in five to ten degrees of ankle dorsiflexion (Figure 2 and 3). The stability of the syndesmosis was assessed by measuring the tibiofibular clear space opening at both the endpoints of the rotational maneuvers and in a neutral ankle position in the axial plane at the level of the AITFL. The measurements were taken on the U.S monitor and then re-evaluated on a hardcopy print. Each measurement was repeated and was averaged if a difference was documented.

Prior to the initiation of the study, reproducibility of the technique was examined by musculoskeletal radiologists in our institute and was found to be comparable with MRI and accurate, as was previously described [4].

Age, gender, degree of activity and leg dominance was recorded. Age group compared were “under 40th group” (under 40 years old) and “over 40th group” (over 40 years old). 110 subjects were studied. There were 59 males and 51 females of mean age 32 years (16–60 years). 60% (66) of subjects were involved in sports in a professional manner. 68 subjects were in the “under 40^th^ group”, and 42 in the “over 40^th^ group”. The mean height was 173 cm (range 149–192), mean TTMM (tibial tuberosity to medial malleolus) length was 35cm (range 30-41cm) and the right leg was dominant in 83% of the subjects. Subjects were classified as “active” or “non-active”. We considered active patients those who trained or participated in sports for more than 15 hours per week, typically professional athletes. Non-active patients were those who participated in sports at a social level or not at all. Height and calf length were also measured. “Calf length” was measured from medial malleolus to tibial tuberosity.

### Statistical analysis

Data was analyzed in relation to age, gender, activity and dominant leg using Pearson correlation, ANCOVA (analysis of covariance) and t-tests. All statistical tests utilized two-sided P values, and the selected level of significance for all variables was alpha = 0.05. SPSS statistical software (version 15.0 SPSS Inc., Chicago) was used to analyze the data.

## Results

**Figure 4:**
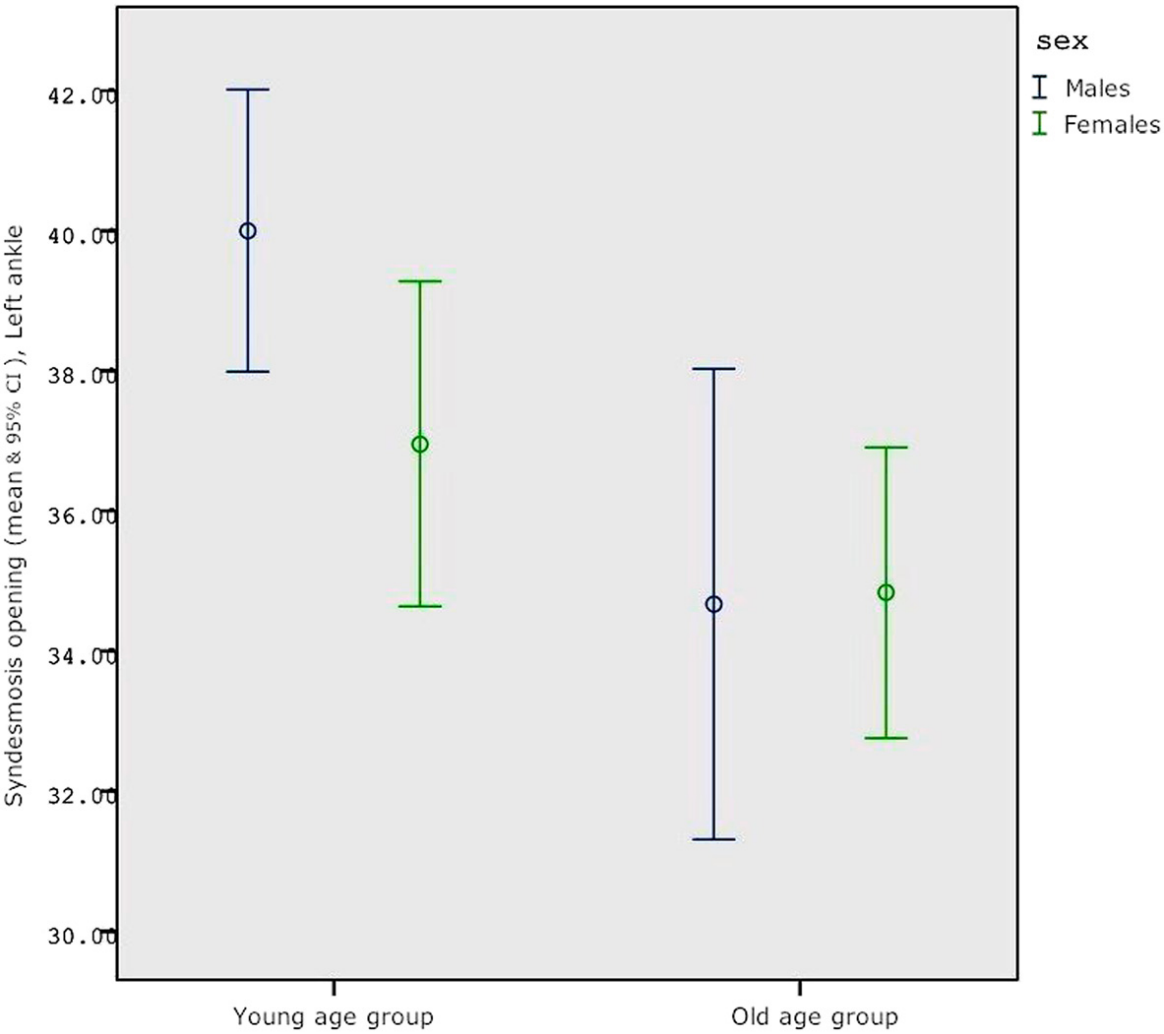
**Absolute AITFL measurements in Neutral position (clear space), Left ankle.** Young age group (under 40 y.o). Older age group (40–60 y.o). The Clear Space absolute measurements are larger with Young age and with males (blue left side lines). This evidence was significant for the age factor (**p<0.005**). The dots represent group means. The value plotted with the dashes at the end of the vertical segments represent the 95% confident interval.

**Figure 5:**
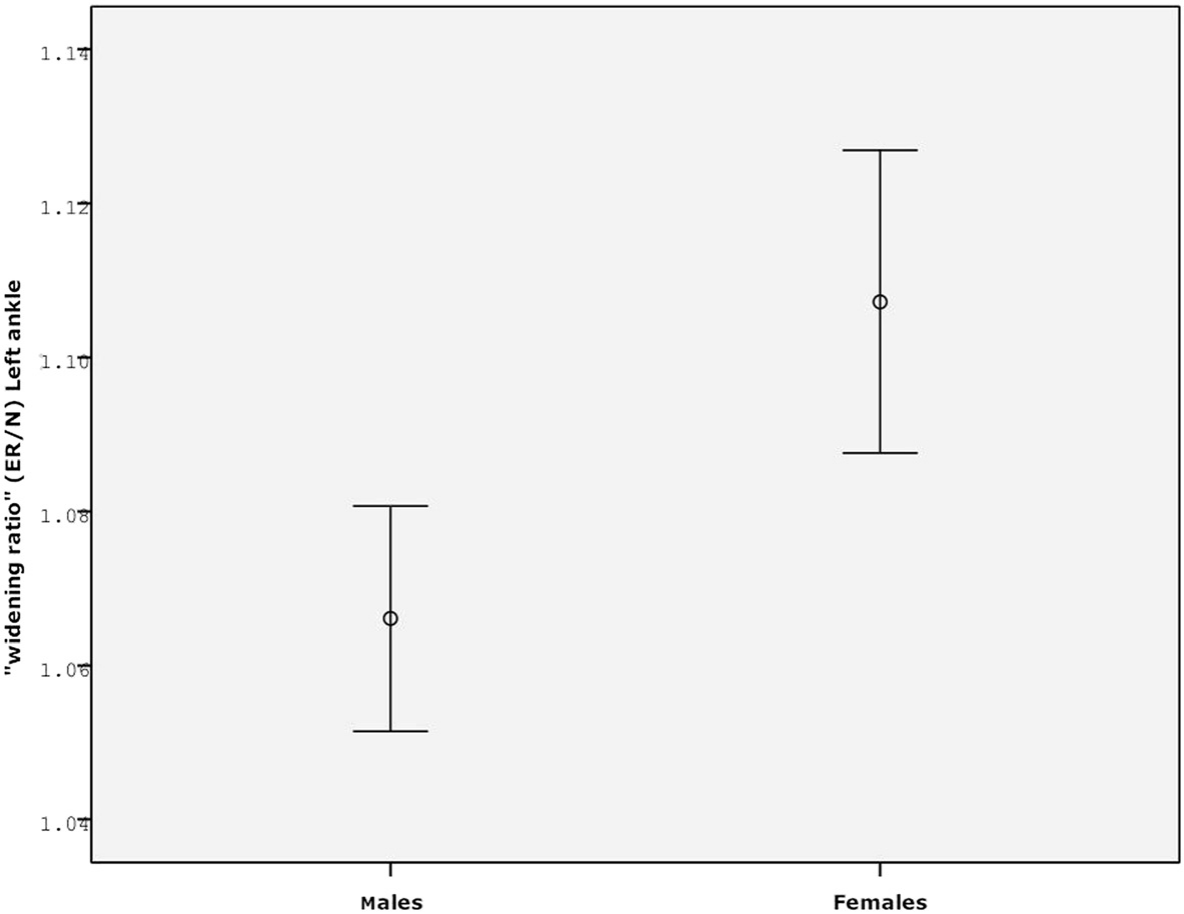
**The “widening ratio” of the joint.** The “widening ratio” of the joint measured as the ER/N ratio in the left ankle. The Females syndesmosis widened under stress more than the male. (p<0.01).

Side to side differences in subjects were not significant in neutral, forced internal and external rotation. The mean opening of the anterior inferior tibio-fibular clear space was N=3.7mm, IR=3.6mm and ER=4.0mm (Values +2SD: 5.06, 4.9, 5.3, Left side, respectively). Therefore the mean increase in anterior clear space with stress application (ER-N) was found to be 0.3 mm. The mean delta ER-IR was 0.4mm, implying the working range of the joint (Table 1). In men and women the clear space was significantly (p<0.05) wider in younger subjects in neutral (Men: Y=3.8 SD-0.6, O=3.4 SD-0.4 \ Women: Y=3.8 SD-0.5, O=3.4 SD-0.4) and with rotational force application (Men ER: Y=4.1 SD-0.6, O=3.6 SD-0.4 \ Women ER: Y=4.1 SD-0.4, O=3.8 SD-0.47) compared to older subjects (Figure 4, Table 2). There was no correlation with activity, height or leg length. The “widening ratio” of the joint was measured as the ratio ER/N. An analysis of covariance (ANCOVA) evaluated this ratio for both sides and gender and with relation to activity status and age. Female subjects had a higher syndesmosis widening ratio with stress application than males (p<0.01). This tended to occur more commonly in active subjects but was not found to be significant (Figure 5).

**Table 1:** Descriptive statistics

	**Min. Age**	**Max. Age**	**Mean**	**Std Dev.**
**Age (years)**	16	60	32	14
	**Min. Height**	**Max. Height**	**Mean**	**Std. Dev.**
**Height (cm)**	149	192	173	8.5
**Position**	**Clear space opening - Min.**	**Clear space opening - Max.**	**Mean**	**Std.Dev**
**Neutral - Lt side**	2.4	5.3	3.78	0.64
**Internal rotation -Lt**	2.1	5.4	3.64	0.63
**External rotation - Lt**	2.69	5.7	4.08	0.63
**Neutral - Rt side**	2.5	5.2	3.73	0.57
**Internal rotation -Rt**	2.4	5.1	3.63	0.57
**External rotation - Rt**	2.8	5.8	4.02	0.57

Measurements in millimeters.

**Table 2:** Group statistics (T-test)

**Position when clear space was measure**	**Group**	**Mean males (mm)**	**Std. deviation (Males)**	**Mean females (mm)**	**Std. deviation (Females)**
**Neutral**	**Y**	3.85	0.61	3.81	0.51
	**O**	3.43	0.42	3.45	0.44
**Internal rotation**	**Y**	3.77	0.62	3.66	0.53
	**O**	3.30	0.42	3.38	0.38
**External rotation**	**Y**	4.10	0.6	4.10	0.49
	**O**	3.66	0.42	3.81	0.47

Y=Young group, O=Old group. All differences (between age groups) are statistically significant.

Measurements in millimeters.

## Discussion

In this study we have established that the normal tibio-fibular clear space is 3.78mm and that the mean widening of the tibio-fibular clear space is 0.3mm for ER-N and 0.44mm for ER-IR on dynamic ultrasound examination. In neutral position a measurement of 5.06 mm (Std. Dev. 0.64, 3.78+2SD=5.06) would be 2 standard deviations above the mean. This suggests a measurement greater than this should be suspected as abnormal. Females had increased clear space opening with forced external rotation, with a significantly greater “widening ratio” of the AITFL to applied stress. This agrees with the literature as female subjects tend to be more flexible than males [7]. There was no correlation between clear space opening and activity, height or leg length. Those who participate in gymnastics and dance tend to have increased joint laxity, [8] however in this study sports activity was not shown to be a significant factor, although a trend was evident.

Younger subjects showed higher absolute clear space opening measurements and stress opening than older subjects, a result which was evident in both genders. This finding is expected given the general increase in stiffness that occurs with aging [9,10]. We also found that activity, height or leg length did not correlate significantly with opening of the joint. That means that tall athletes, for an example, would not have a larger opening than younger non-active females.

The results of a previously published study indicate that a dynamic ultrasound examination can accurately diagnose a syndesmotic injury in cases of latent (not evidenced on radiograph films) grade 3 AITFL sprains [4]. Similar to other ligament injuries, injuries to the AITFL are graded based on stretch (grade1), partial tear (grade 2), or complete rupture (grade 3) [11]. Complete rupture of the AITFL is described as a stage one syndesmotic injury, which with additional force may cause the talus to spread the tibia and fibula apart. This can subsequently damage the posterior aspect of the syndesmosis (PITFL and transverse ligament) and cause a stage three injury [12] or a frank fracture.

One of the strengths of this study is that it provides information regarding the width of the anterior tibio-fibular clear space in a neutral unloaded position and also provides dynamic assessment of the opening of this space with the application of a deforming force. We believe this is the first study to record syndesmotic opening dynamically in normal subjects.

One of the features of this presented method is reproducibility. Ultrasound examination is operator dependent and requires regular practice to be familiar with localized imaging and normal values. We have used a published standardized technique to measure the width of the anterior tibio-fibular clear space [4]. In this series one experienced radiologist (XX) performed the US examinations accompanied by a muscloskeletal ultrasound trained sports physician (YY) in an attempt to minimize intra-observer error. Prior to the initiation of the study, reproducibility was verified by two other musculoskeletal radiologists in our institute. The technique of ultrasound measurement of the anterior clear space was quick to learn and was considered to be reproducible, comparable with MRI and shown to have an accuracy of 89-100% for AITFL injury [4]. By recording the values of syndesmotic opening in normal subjects we have provided normal reference values, which can assist less experienced musculoskeletal ultrasonographers. It is important that all measurements are taken at the same level above the ankle joint in order to standardize the relation between N, ER and IR positions. Even if the probe is placed a few millimeters higher, the absolute clear space opening measurement in neutral position might be increased but dynamic evaluation will keep the same relationships.

Another potential weakness is the lack of a standardized deforming force to recreate the opening of the syndesmosis. As this was a clinical study on live subjects the syndesmosis was not tested to failure but merely the width at maximal opening was determined. External rotation was performed until a firm recognizable end point was reached. Error was minimized by the same examiner applying a similar rotational force, and appreciating the same firm end point from neutral dorsiflexion/plantarflexion in each case.

Age groups compared in this study were under and over 40 years of age. We have chosen 40years as a cutoff age between young and older group as most of our study subjects fell into these two main groups. We had one cluster around 20–30 years of age, which usually stand for the population who tend to suffer from syndesmosis injuries, and another cluster around the 50 years of age. We had very few subjects between 35 to 45 years of age, and no one at that exact age.

The kinematic biomechanics of the distal tibio-fibular syndesmosis are subtle and are coupled with the ankle joint. With ankle dorsiflexion the talus rotates about 5 degrees externally about it’s axis in dorsiflexion and internally with plantarflexion [13,14]. Correspondingly the fibula also rotates 3–5 degrees externally about its axis in the fibular groove of the tibia with a full range of ankle motion [13,15]. Norkus and Floyd [15] established that with fibula rotation the syndesmosis widens by 1–2 mm when the foot is moved from plantar to dorsiflexion [15].

Given the subtle movements of the intact syndesmosis it is not surprising that clinical detection of syndesmotic instability is difficult. The calf compression test (squeeze test) [2], the External Rotation test [1], and the direct eversion maneuver have all been described to clinically detect syndesmotic instability, with sensitivity ranging from 33-92% [3]. Imaging therefore plays a key role in the detection of syndesmotic injury.

AP and Mortise plain static radiographs have shown sensitivity, specificity, and accuracy of 44.1%, 100%, and 63.5% for the AP view and 58.3%, 100%, and 71.2% for the mortise view when compared to arthroscopic examination [16]. Nielson et al. [17] found no association between the tibio-fibular clear space and overlap measurements in patients with syndesmotic injuries on plain radiographs when compared to MRI. Since the width of the tibio-fibular clear space does not change with rotation and the tibio-fibular overlap, width of the tibia and fibula, and medial clear space all do change with rotation comparison views may warranted to account for biologic variation [5,18,19].

CT scanning is sensitive for bony alignment and can identify 2–3 mm diastases not seen on a plain radiograph [20]. Spiral CT scan analysis reveals that a 1 mm diastasis increases the syndesmosis volume by approximately 43% with an additional 20% for each subsequent millimeter [21].

MRI is considered the optimal static imaging modality for AITFL integrity, but cost and availability do not warrant use for all suspected syndesmotic injuries [6,22,23]. There is potential for the development of dynamic MR imaging to visualize ligament laxity and joint instability in the future.

Direct arthroscopic visualization of clear space opening and syndesmotic instability may be considered to be the gold standard but is invasive and not without risks. Milz et al. [6] compared static ultrasound examination and MRI studies for the examination of the lateral ligaments complex and tibio-fibular syndesmosis. Ultrasound was found to have a sensitivity of 66% and specificity of 91% for AITFL injury.

In a previous study assessing ultrasound imaging of the syndemsosis in AITFL injured patients [4], the syndesmosis was found to widen as much as 1.1mm from neutral to external rotation and 1.85mm moving from external to internal rotation, 11 and 9 times greater than in normal joints which has movement of this 0.1 mm and 0.2 mm in respectively., In comparing these two studies it is evident that healthy syndesmoses widen significantly less than traumatized syndesmoses with AITFL injury. This demonstrates the potential of ultrasound examination in determining the integrity of the syndesmosis following injury.

The control healthy patients of the previous published study showed a normal small opening between the anterior fibula and tibia at the syndesmosis level with mean (ER-IR) of 0.2 mm. These values are similar to the findings of this series.

In the previous study a minimal value of 0.4mm for increased opening of the joint clear space with ER-N had a sensitivity and specificity of 89% for AITFL injury. This cut off is reinforced by this current study where a maximal opening of the normal clear space was found to be 0.3mm.

Using dynamic ultrasound examination we have determined the width and opening of the anterior tibio-fibular clear space in neutral and with forced external and internal rotation positions, Allowing radiologists and sports physicians to determine abnormal syndesmotic laxity and AITFL injury in a readily accessible, reproducible and radiation free imaging modality.

## Conclusions

Normal values for the syndesmosis clear space on ultrasound examination were determined as 3.78 mm in neutral, 3.64 mm in internal rotation and 4.08mm in external rotation. The clear space was shown to decrease with age both as an absolute measure and when rotational stresses are applied. Females tend to have a larger clear space and a greater functional widening.

These findings provide a useful reference for radiologists and sports physicians when performing ultrasound assessment of ankle syndesmotic injuries as well as a measurable tool for diagnosis of this often missed injury and we encourage use of this modality.

## Competing interest

The authors declare that they have no competing interest.

## Authors’ contributions

OMD is the principal investigator, initiator of this study and was involved in logistical setup and drafting the manuscript. MC was involved in statistical analysis and drafting the manuscript. LL was involved in logistical setup, data recording and collection and literature review. MN was involved in patient recruitment and study design. HK was involved in data recording and collection. GM was involved in patient recruitment and study design. BC was involved in statistical analysis and sonographic data evaluation. EK performed the sonographic testing and evaluations. All authors read and approved the final manuscript.

## Pre-publication history

The pre-publication history for this paper can be accessed here:

http://www.biomedcentral.com/2052-1847/5/9/prepub
